# Editorial: Molecular and physiological aspects of sarcopenia in the older person: mechanisms, diagnostics and therapy

**DOI:** 10.3389/fmed.2023.1330893

**Published:** 2024-01-03

**Authors:** Guilherme Eustáquio Furtado, Marco Vincenzo Narici, Tzvi Dwolatzky

**Affiliations:** ^1^Polytechnic Institute of Coimbra, Applied Research Institute, Coimbra, Portugal; ^2^Research Centre for Natural Resources Environment and Society (CERNAS), Polytechnic Institute of Coimbra, Coimbra, Portugal; ^3^Department of Biomedical Sciences, Institute of Physiology, University of Padua, Padua, Italy; ^4^MRC-ARUK Centre for Musculoskeletal Ageing, University of Nottingham, Derby, United Kingdom; ^5^Department of Biomedical Sciences for Health, University of Milan, Milan, Italy; ^6^Geriatric Unit, Rambam Health Care Campus, Haifa, Israel; ^7^The Ruth and Bruce Rappaport Faculty of Medicine, Technion–Israel Institute of Technology, Haifa, Israel

**Keywords:** sarcopenia, geriatric research, inflammaging, muscle mass assessment, aging and muscle health, muscle atrophy mechanisms

In the first article of our exploration of the most recent research findings in the Molecular and Physiological Aspects of Sarcopenia in the Older Person RT, we highlight a significant connection between sarcopenia and serum Follicle-Stimulating Hormone (Ke et al.). As we delve into the domain of sarcopenia-related research, our attention is drawn to a study that examines the use of Xanthine Oxidoreductase (XOR) inhibitors and their potential impact on sarcopenia among patients undergoing hemodialysis (HD). Sarcopenia is a prevalent concern in this population, and this second study delves into the role of XOR inhibitors in addressing this condition (Kurajoh et al.). Indeed, there is a concerted effort to identify novel biochemical markers associated with sarcopenic conditions (1), as early detection, facilitated by precise diagnoses, can enhance the efficacy of comprehensive treatment approaches (2).

In the current quest to uncover the complexities of sarcopenia, we turn our attention to a groundbreaking study that delves into the underlying mechanisms and immunological profiles related to this condition (Abdelrahman et al.). Continuing our exploration, we shift our focus to a captivating study conducted in the agricultural and pastoral regions of China. This research unraveling the relationship between plasma tumor necrosis factor-α, and their association with sarcopenia among community-dwelling older adults (Wumaer et al.). The inspection of the intricate relationship between aging, physical activity (PA), and inflammation continues with a study focusing on inflammaging in physically active older women. This research delves into how inflammaging relates to body composition and physical factors, shedding light on this complex interplay (Santos et al.). In detail, these lastthree studies serve as an invitation for readers to scrutinizing the molecular mechanisms that underpin the connection between inflammation and muscle health (3), as well as exploring potential interventions aimed at ameliorating the impact of inflammation on sarcopenia (4).

Regular PA and exercise are cornerstones of healthy aging, especially for those in long-term care facilities. In our pursuit of advancing the health and wellbeing of older individuals, we now turn our attention to a promising approach: exergame-based exercise. This innovative method has the potential to address muscle loss, muscle strength, cognition, and functional performance among older adults residing in rural long-term care facilities (Tuan et al.). Screening and early detection of sarcopenia is essential for timely intervention and improved outcomes. In this study, we delve into the prospective relationship between physical performance tests and the risk of developing sarcopenia in individuals aged 55 and over in Malaysia. The research focuses on determining suitable cut-off values for screening activities and highlights the importance of early detection (Megasari et al.).

In fact, the early diagnosis of sarcopenia among community-dwelling older individuals is of paramount importance (2). Through timely identification, healthcare providers can implement multimodal interventions that encompass dietary re-education, targeted supplementation, and structured exercise regimens (4, 5). This “gold standard” approach not only addresses the progression of sarcopenia, and reduce the risk of adverse outcomes such as hospitalization, institutionalization, and premature mortality (6–8).

Regarding to unlock the secrets of longevity and vitality in aging, we arrive at a crucial study that explores the remarkable effects of caloric restriction (CR) on age-related muscle atrophy. Sarcopenia has been a pressing concern in geriatric research, and understanding the mechanisms behind CR's influence is of paramount importance (Lv et al.). In the continuing exploration of sarcopenia and its clinical implications, we shift our focus to a novel and cost-effective tool for assessing muscle mass and its prognostic value in hospitalized patients. This meta-analysis delves into the relationship between the creatinine/cystatin C ratio and sarcopenia, providing valuable insights into the utility of this biomarker (Zheng et al.). These two studies collectively contribute to our knowledge of the mechanisms involved in sarcopenia and highlight the significance of proper nutrition in maintaining muscle health in the older population (9). They underscore the importance of early detection and interventions to improve the nutritional status and overall wellbeing of older individuals affected by sarcopenia.

The link between muscle health and coronary heart disease is a critical area of investigation, as it can have profound implications for the wellbeing of patients. This study that delves into circulatory biomarkers associated with low muscle mass in individuals with coronary heart disease. These findings provide insights into the mechanisms of muscle health in this specific population (James et al.). In our continuing, we now turn our attention to a study conducted in Brazil, which investigates the prevalence and interplay of obesity, sarcopenia, and metabolic syndrome. These conditions are of critical concern in the aging population, but understanding their prevalence and associations is essential for effective intervention (Pinheiro et al.). These insights are crucial to enhance cardiovascular health and wellbeing in aging individuals. Furthermore, this understanding is essential for addressing the sarcopenic obesity (10), where individuals experience the double burden of muscle loss and excess fat, putting them at higher risk for cardiovascular issues.

In summary, this RT provided a comprehensive and illuminating exploration of diverse facets of aging-related research. It has successfully elucidated critical links between aging, muscle health, and a myriad of related factors, ranging from molecular mechanisms to clinical interventions. The Research Topic of studies within this issue has shed light on the complex interplay of inflammation, exercise, nutrition, and biomarkers, offering valuable insights for the field of sarcopenia-related research.

As a contemporary reflection, this RT is not only a cornerstone in advancing our understanding of the aging process but also plays a central role in addressing the Sustainable Development Goals (SDGs) by United Nations (11). Through emphasizing the significance of early detection and intervention, it contributes significantly to SDG 3 (Good Health and Wellbeing) by promoting the health and wellbeing of older adults. In addition to the objectives of this RT, it's essential to highlight that the knowledge presented here is in harmony with the global commitment to attaining Sustainable Development Goal 4 (SDG 4)—Quality Education (12). The wealth of knowledge presented herein not only expands the horizons of geriatric and gerontology research but also paves the way for a brighter, healthier future for our aging population, aligning with SDG 10 (Reduced Inequalities) by focusing on the specific needs of older individuals and ensuring inclusivity. Additionally, this research supports SDG 17 (Partnerships for the Goals) by fostering collaboration among researchers, healthcare providers, and policymakers to collectively address the challenges of aging and promote healthier aging globally.

## Author contributions

GF: Conceptualization, Project administration, Writing—original draft, Writing—review & editing. MN: Conceptualization, Project administration, Writing—original draft. TD: Conceptualization, Project administration, Supervision, Writing—original draft.
